# Prevalence and Assessment of Knowledge, Attitudes, and Practices of Tobacco Use Among Medical and Dental Students in Bhubaneswar, Odisha

**DOI:** 10.7759/cureus.58617

**Published:** 2024-04-19

**Authors:** Nancy Satpathy, Himanshu S Pradhan, Swapna Sarangi, Venkatarao Epari, Pratap K Jena, Samarendra Dash, Debi P Mohanty, Pratisha Mishra

**Affiliations:** 1 Community Medicine, Institute of Medical Sciences and SUM Hospital, Siksha 'O' Anusandhan, Bhubaneswar, IND; 2 Non-communicable Division III, Indian Council of Medical Research, New Delhi, IND; 3 Public Health, Kalinga Institute of Industrial Technology, Bhubaneswar, IND; 4 Medicine and Surgery, Lady Hardinge Medical College, New Delhi, IND; 5 Health Care Management, Swiss School of Business and Management Geneva, Geneva, CHE; 6 Orthodontics and Dentofacial Orthopaedics, National Aluminium Company Limited, Bhubaneswar, IND; 7 Oncology, Institute of Medical Sciences and SUM Hospital, Siksha 'O' Anusandhan, Bhubaneswar, IND; 8 Public Health Dentistry, Sardar Patel Post Graduate Institute of Dental and Medical Sciences, Lucknow, IND

**Keywords:** tobacco cessation, health profession students, ghpss, tobacco, current use

## Abstract

Background

Tobacco use remains a significant global public health concern, causing millions of preventable and premature deaths annually and imposing substantial economic burdens. India, the second-largest producer and consumer of tobacco products worldwide, bears a significant burden of tobacco-related morbidity and mortality. Medical and dental students represent the future healthcare workforce and role models; hence, their tobacco consumption and attitude would play a vital role in tobacco control. This study aims to estimate the prevalence and assess the knowledge, attitudes, and behaviors regarding tobacco use among medical and dental students in Bhubaneswar, Odisha.

Methods

A descriptive cross-sectional study was conducted using the Global Health Professional Students Survey (GHPSS) questionnaire. The study included third-year Bachelor of Medicine and Bachelor of Surgery (MBBS) and Bachelor of Dental Surgery (BDS) students from two private medical and two dental colleges in Bhubaneswar, Odisha. Data were collected from February to April 2019 through anonymous self-administered questionnaires, and descriptive and bivariate analyses were performed.

Results

A total of 400 students were surveyed, with 16% reporting being current smokers (24.3% males, 8.7% females). Furthermore, 36.8% had tried cigarettes and other tobacco products. Nonsmokers demonstrated stronger support for comprehensive tobacco control policies, such as banning advertising and smoking in public places, compared to current smokers. Most students acknowledged the importance of recording tobacco use history and providing educational materials; however, only around 40% had received formal training on smoking cessation.

Conclusion

The findings highlight the need for targeted intervention among medical and dental students for tobacco cessation. It is vital to foster a positive attitude toward tobacco control among future healthcare professionals. Health professional institutions should take proactive steps to prevent tobacco use among students and develop initiatives to motivate successful cessation training. Investing in tobacco control education for healthcare professionals is crucial to empower them in tobacco cessation efforts and promote healthier societies.

## Introduction

Tobacco use remains a significant global public health concern, posing a threat to individuals' well-being and imposing substantial societal burdens [1]. Despite widespread awareness of its detrimental effects, tobacco use continues to impose a heavy toll, with millions of lives lost annually to tobacco-related diseases [2]. According to the WHO, tobacco use is responsible for more than eight million deaths annually worldwide, making it one of the leading causes of preventable death [3]. The impact of tobacco use extends beyond the loss of life, encompassing a wide range of health, economic, and social consequences. In addition to causing a myriad of health problems, including cardiovascular diseases, respiratory illnesses, and various forms of cancer, tobacco use also imposes a significant economic burden on individuals, families, and healthcare systems [4,5]. The costs of treating tobacco-related diseases, coupled with the economic toll of lost productivity and premature deaths, amount to billions of dollars each year [6]. This immense financial burden has already overstretched healthcare systems and impeded economic growth and development.

The prevalence of tobacco use varies widely across different regions and demographic groups, reflecting complex interactions between individual behavior, sociocultural factors, and public health policies [7]. While tobacco use rates have declined in some high-income countries because of effective tobacco control measures, the tobacco epidemic continues to grow in many low- and middle-income countries, where tobacco companies aggressively target vulnerable populations with their marketing tactics [8].

India, home to one of the world's largest populations, bears a significant burden of tobacco-related morbidity and mortality [9]. Despite efforts to curb tobacco use, India continues to have one of the highest rates of tobacco consumption globally, with millions of people addicted to tobacco in various forms, including cigarettes, bidis, and smokeless tobacco [9]. The situation is particularly alarming among young people, including medical and dental students, who represent the future healthcare workforce [10].

Recognizing the urgent need to address the tobacco epidemic, governments, public health organizations, and civil society groups have implemented a range of tobacco control measures aimed at reducing tobacco consumption and preventing initiation among young people [11]. These measures include tobacco taxation, smoke-free policies, bans on tobacco advertising and promotion, and support for tobacco cessation services [12]. However, the effectiveness of these interventions hinges on their implementation and enforcement, as well as the engagement of key stakeholders, which include healthcare professionals [13].

Healthcare professionals, including medical and dental students, play a crucial role in tobacco control efforts, serving as frontline advocates, educators, and providers of cessation support. As future healthcare providers, medical and dental students have a unique opportunity to influence tobacco-related behaviors and attitudes among their peers and patients, thereby contributing to the broader effort to create tobacco-free environments and promote public health [14]. Odisha, located in eastern India, exhibits a higher prevalence rate of tobacco use among individuals aged 15 and above, with rates at 45.6%, including 57.6% and 33.6% among men and women, respectively [15]. The abundant healthcare institution presence in Bhubaneswar offers a conducive environment for the study. Against this backdrop, this study aims to estimate the prevalence and assess the knowledge, attitudes, and behaviors regarding tobacco use among medical and dental students in Bhubaneswar, Odisha.

## Materials and methods

In a descriptive cross-sectional study, out of three private medical colleges and three dental colleges in Bhubaneswar, two private medical and two dental colleges were included. This study was conducted from February to April 2019. The study included all the third-year Bachelor of Medicine and Bachelor of Surgery (MBBS) and Bachelor of Dental Surgery (BDS) students of the respective medical and dental colleges.

The study employed a standardized, adapted, and validated version of the Global Health Professional Students Survey (GHPSS) 2005 India questionnaire as part of the Global Tobacco Surveillance System (GTSS) advocated by the WHO, the US Centers for Disease Control and Prevention (CDC), and the Canadian Public Health Association [16]. Because of the significant time lapse since its original use, adaptations were made to this questionnaire. These adaptations included adding questions about local forms of tobacco and removing irrelevant questions, resulting in a comprehensive questionnaire comprising 42 questions. Subsequently, the adapted GHPSS questionnaire underwent rigorous face and content validation processes to ensure its reliability and validity.

The questionnaire underwent a pilot study involving 20 students to assess its comprehensibility and the respondents' ability to answer without assistance. The pilot study revealed a prevalence rate of currently smoking cigarettes at 12.26% overall in medical students, which is comparable to the GHPSS 2005 India prevalence rate of 11.6%. Among dental students, the prevalence rate was 10.23%, also similar to the GHPSS 2005 India rate of 9.6% for current smoking of cigarettes.

The participants were informed about the voluntary nature of participation and their right to withdraw at any time, for any reason, before data collection. After obtaining informed and written consent from the participants, the anonymous questionnaire was administered during regular class sessions by a single investigator and collected on the same day. No incentives or inducements were provided to participants during the study, and the confidentiality of the information was assured. Ethical approval for the study was obtained from the institutional review committee of the KIIT School of Public Health, KIIT University (Letter No.: Ref. No.: KIMS/KIIT/IEC/609/2019).

The current smokers were defined as those who smoked cigarettes daily or occasionally in the past 30 days before the survey. Nonsmokers were defined as those who have never smoked a cigarette in their lifetime. Ever smokers were described as those who have, in their lifetime, smoked even a single cigarette. Additionally, other tobacco products, such as chewing tobacco (khaini, gutkha, pan masala, zarda), snuff, bidis, hookahs, cigars, or pipes, were defined. The outcome measure was “being a current smoker,” defined as those who smoked cigarettes at least one day during the 30 days before the survey. Knowledge was defined as the expertise and skill acquired during the course curriculum among third-year MBBS and BDS students, knowledge of the harmful effects of smoking, and the role and responsibilities of health professionals. Attitude was defined as feeling, acting, or expressing an opinion toward tobacco. The term "behavior" was defined as the way a student behaved toward tobacco use.

Descriptive and bivariate analyses were performed using Statistical Product and Service Solutions (SPSS, version 20; IBM SPSS Statistics for Windows, Armonk, NY). Statistical significance was considered at a P value < 0.05.

## Results

A total of 400 students were surveyed, of whom 177 (44.29%) were males. The mean age of the participants was 21.39 years, with a standard deviation of ±1.39 years.

Table 1 presents the prevalence of tobacco use among the participants, shedding light on their smoking behaviors and experiences with various tobacco products. About 16% (n=63) of the participants reported being current smokers, which was notably higher among males (24.3%) compared to females (8.7%). Among the surveyed individuals, 147 (36.8%) participants reported ever trying or experimenting with smoking cigarettes. The prevalence varied across genders, with more males (n=88, 49.7%) compared to females (n=59, 26.5%) having tried cigarettes. Regarding the age of initiation for smoking cigarettes, the majority of the participants (n=62, 15.6%) tried their first cigarette between the ages of 11 and 19, with male predominance (n=41, 23.2%) compared to females (n=21, 9.3%). Additionally, the data revealed that 147 (36.8%) participants had ever tried other tobacco products, such as chewing tobacco, snuff, bidis, hookahs, cigars, or pipes. Among them, 23 (5.7%) were currently using other tobacco products (Table 1).

**Table 1:** Prevalence of tobacco use among medical and dental students (n=400)

Variable		Female (N=223), n (%)	Male (N=177), n (%)	Total (N=400), n (%)
Have you ever tried or experimented with cigarette smoking?	Yes	59 (26.5)	88 (49.7)	147 (36.8)
	No	164 (73.5)	89 (50.3)	253 (63.8)
If yes, what was your age when you first tried a cigarette (in completed years)?	≤10	5 (2.2)	6 (3.4)	11 (2.8)
	11-19	21 (9.3)	41 (23.2)	62 (15.6)
	20-24	31 (13.9)	41 (23.2)	72 (18)
Are you currently a cigarette smoker?	Yes	20 (8.7)	43 (24.3)	63 (15.75)
	No	203 (91)	134 (75.7)	337 (84.3)
Have you ever tried other tobacco products?	Yes	59 (26.5)	88 (49.7)	147 (36.8)
	No	164 (73.5)	89 (49.7)	253 (63.8)
Are you currently using other tobacco products?	Yes	9 (3.9)	14 (7.9)	23 (5.7)
	No	214 (96.1)	163 (92.1)	377 (94.3)

The majority of both noncurrent smokers and current smokers reported being taught about the dangers of smoking (86.4% and 85.7%, respectively) during class. A significant difference was observed between the two groups in being taught about the reasons why people smoke during class (70% noncurrent smokers vs. 55.6% current smokers, p=0.039). No significant difference was observed between the two groups in acknowledging the importance of recording tobacco use history (86.6% noncurrent smokers, 82.5% current smokers, p=0.428). However, no significant differences were observed between noncurrent smokers and current smokers in terms of receiving formal training on smoking cessation (40.7% vs. 36.5%, p=0.577), awareness of nicotine replacement therapies (92.3% vs. 88.9%, p=0.33), or familiarity with antidepressant use in tobacco cessation programs (62.3% vs. 55.6%, p=0.326) (Table 2).

**Table 2:** Knowledge of tobacco cessation in medical school curriculum among medical and dental students * means statistically significant at p value <0.05; ** means statistically significant at P value <0.01; and *** means statistically significant at p value ≤0.001.

Knowledge		Nonsmoker	Current smoker	P value
		No, n (%)	Yes, n (%)	
Were you taught during class about the dangers of smoking?	Yes	291 (86.4)	54 (85.7)	0.844
	No	46 (13.6)	9 (14.3)	
Were you taught during class about the reasons why people smoke?	Yes	235 (70)	35 (55.6)	0.039*
	No	102 (30)	28 (44.4)	
Have you learned that it is important to record tobacco use history?	Yes	292 (86.6)	52 (82.5)	0.428
	No	45 (13.4)	11 (17.5)	
Have you ever received formal training on smoking cessation?	Yes	137 (40.7)	23 (36.5)	0.577
	No	200 (59.3)	40 (63.5)	
Have you learned that it is important to provide educational materials to support smoking cessation to patients who want to quit smoking?	Yes	259 (76.9)	46 (73)	0.521
	No	78 (23.1)	17 (27)	
Have you ever heard of nicotine replacement therapies?	Yes	311 (92.3)	56 (88.9)	0.33
	No	26 (7.7)	7 (11.1)	
Have you heard of antidepressant use in tobacco cessation programs (such as bupropion or Zyban)?	Yes	210 (62.3)	35 (55.6)	0.326
	No	127 (37.7)	28 (44.4)	

Nonsmokers demonstrated significantly stronger support for comprehensive tobacco control policies compared to current smokers. They were more likely to endorse a complete ban on advertising tobacco products (88.1% vs. 84.1%, p=0.001) and advocate for implementing smoking bans in various settings, such as restaurants (93.8% vs. 76.2%, p<0.001), discos/bars/pubs (76.3% vs. 34.9%, p<0.001), and all enclosed public places (95% vs. 85.7%, p=0.012). Both groups largely agreed that health professionals should obtain specific cessation training (97.6% nonsmokers vs. 87.3% current smokers, p=0.001) and that they serve as role models (90.2% vs. 81%, p=0.048). While nearly all nonsmokers felt health professionals should routinely advise quitting smoking (97.3% vs. 88.9%, p=0.006) and other tobacco products (97.3% vs. 82.5%, p<0.001), the percentages were lower among current smokers. There were no significant differences regarding whether health professionals are involved in advising about smoking cessation (p=0.322), the chance of quitting increasing with their advice (p=0.272), or whether smoking health professionals are less likely to advise patients to stop smoking (p=0.142). However, significantly more nonsmokers (87.24%) than current smokers (61.9%, p<0.001) felt health professionals using tobacco products were less likely to advise patients to stop smoking (Table 3).

**Table 3:** Attitudes and behavior toward tobacco control policies and smoking cessation among medical and dental students * means statistically significant at P value <0.05; ** means statistically significant at P value <0.01; and *** means statistically significant at P value ≤0.001.

Attitudes and behavior		Nonsmoker	Current smoker	P value
		No, n (%)	Yes, n (%)	
Should tobacco sales to adolescents be banned?	Yes	289 (86.3)	53 (84.1)	0.693
	No	46 (13.7)	10 (15.9)	
Should the advertising of tobacco products be banned entirely?	Yes	297 (88.1)	44 (84.1)	0.001^***^
	No	40 (11.9)	19 (15.9)	
Should smoking be banned in restaurants?	Yes	316 (93.8)	48 (76.2)	<0.001^***^
	No	21 (6.2)	15 (23.8)	
Should smoking be banned in discos/bars/pubs?	Yes	257 (76.3)	22 (34.9)	<0.001^***^
	No	80 (23.7)	41 (65.1)	
Should smoking in all enclosed public places be banned?	Yes	320 (95)	54 (85.7)	0.012^*^
	No	17 (5)	9 (14.3)	
Should health professionals get specific training on cessation techniques?	Yes	329 (97.6)	55 (87.3)	0.001^***^
	No	8 (2.4)	8 (12.7)	
Do health professionals serve as “role models” for their patients and the public?	Yes	304 (90.2)	51 (81)	0.048*
	No	33 (9.8)	12 (19)	
Should health professionals routinely give advice on quitting smoking?	Yes	328 (97.3)	56 (88.9)	0.006**
	No	9 (2.7)	7 (11.1)	
Should health professionals routinely give advice on quitting other tobacco products?	Yes	323 (95 .8)	52 (82.5)	<0.001^***^
	No	13 (4.2)	11 (17.5)	
Do health professionals have a role in advising about smoking cessation?	Yes	324 (96.1)	59 (93.7)	0.322
	No	13 (3.9)	4 (6.3)	
Does the chance of quitting smoking increase if a health professional gives advice?	Yes	283 (84)	49 (77.8)	0.272
	No	54 (16)	14 (22.2)	
Are health professionals who smoke less likely to advise patients to stop smoking?	Yes	284 (84.3)	48 (76.2)	0.142
	No	53 (15.7)	15 (23.8)	
Are health professionals who use tobacco products (e.g., chewing tobacco, snuff, bidis, hookahs, cigars, or pipes) less likely to advise the patient to stop smoking?	Yes	294 (87.24)	39 (61.9)	<0.001^***^
	No	43 (12.75)	24 (38.1)	

## Discussion

This study was done to estimate the prevalence of tobacco use and the extent of knowledge, attitude, and behavior of tobacco usage among health professional students in Bhubaneswar, Odisha.

The current study revealed that approximately one-third of medical and dental students have used cigarettes at least once in their lives. This finding contrasts with those of Boopathirajan et al. [17] and Lam et al. [18], whose studies indicated lower rates of cigarette exposure among students (10.9% and 7.2%, respectively). In contrast, Sreeramareddy et al. [14], Armstrong et al. [19], and Rodakowska et al. [20] reported higher percentages of students with access to smoking (29.2%, 38.7%, and 42%, respectively).

According to our survey, 15.8% of students were current smokers, of which 9% were females and 24.3% were males. It was observed that if the distinction between female and male students is made, male students exceed female students, contributing to current smokers to a higher number. Furthermore, our analysis highlighted the impact of smoking accessibility within college premises as a critical variable influencing smoking prevalence, as discussed in prior research [17,21-23].

Our current study showed that more than 90% of the students preferred banning the sale of tobacco products to adolescents, prohibiting the advertisement of tobacco products, and banning smoking in restaurants and public places. This was one of the positive aspects of students’ attitudes toward the use and prevalence of tobacco, which is one of the massive emerging pandemics marching forward persistently. One more positive view of the current study revealed that nearly three-fourths of the students reported that smoking should be banned in discos/bars/pubs. Contrary to the above two points of the study, researchers like Chand et al. [24], Mohan et al. [25], and Sreeramareddy et al. [14] have contributed their studies toward similar studies that acted as corroborations for the facts of the current study. It was revealed that, as per our current study, although the stated percentage of students favored banning, on the flip side, only three-fourths of the students believed that tobacco products should be banned in discos, bars, and pubs. This study also stated that nearly three-fourths of the students felt that healthcare professionals should be role models for their patients.

Refraining from the prevalence and use of this severe problem of tobacco use was one of the critical factors for students. Our study showed that approximately one-third of students have made efforts to curb this dreadful habit. Additionally, 10.35% of smokers have also received counseling and suggestions to stop smoking cigarettes, and more than 80% of medical practitioners are unlikely to suggest their patients quit smoking, which is lower than the 87.2% reported in a study by Muza et al. [26], which showed that moderately high proportions contribute around 87.2%. In an analogy to the above-stated study reports, more than 50% of medical students have attempted to stop smoking. To the point of counseling, some studies reported that more than half of smokers had received recommendations for quitting smoking, which acted as authenticated resources for the current study [27,28].

Our study found that 40% of the students endorsed formal skills and training for smoking cessation among patients, whereas 76.8% were educated that providing educational materials to patients is essential. Saulle et al. [29] found that 87.7% of students were in the category of receiving training, and 58.5% were informed about providing study materials to patients.

Our study also found that there were students who had already perceived the use of nicotine replacement therapies in tobacco cessation programs, and this contributed to more than one-fourth of correspondents. In contrast, more than half were already under the impression of the use of antidepressants in tobacco cessation programs. Regarding antidepressants in tobacco cessation, researchers like Jradi et al. have contributed data that acted as solid evidence for our study, which had a slightly higher proportion (i.e., 63%), where students are already enlightened about the use of antidepressants in tobacco cessation programs [30].

The study's limitation lies in its reliance on self-reported data from participating students. This method introduces the potential for reporting bias, where students may not accurately report their tobacco use habits or attitudes. Additionally, the study's focus on a specific geographical area and a limited number of medical and dental colleges in Bhubaneswar, Odisha, makes generalizing the findings to a broader population of medical and dental students challenging.

Future research could explore strategies to minimize reporting bias in self-reported data and expand the study to include a more diverse and representative sample of medical and dental students from various regions. Additionally, conducting longitudinal studies could provide valuable insights into changes in tobacco use behaviors and attitudes over time among this population.

## Conclusions

The findings of this study underscore the critical role of healthcare professionals, including doctors, nurses, educators, and other health advocates in implementing comprehensive anti-tobacco and tobacco cessation measures. Given the devastating impact of tobacco use on individuals and communities, tobacco control and cessation activities remain crucial public and personal health priorities. It is imperative that health professional institutions, along with associations committed to public health and education, take proactive steps to prevent tobacco use among health professionals. Collaborative efforts are needed to develop and implement initiatives that motivate all health professionals to undergo successful cessation training.

The GHPSS has highlighted a significant unmet need for cessation assistance among students and technical skill gaps in providing practical support to potential patients. While the GHPSS offers valuable insights into tobacco behavior and attitudes among health professional students, further work is necessary to strengthen the evidence base for effective tobacco-related curricula, particularly in diverse cultural and economic settings. Investing in enhancing tobacco control education for healthcare professionals is essential for substantial reductions in tobacco product use. By prioritizing resources toward improving the quality of tobacco control education, we can empower health professionals to play a pivotal role in tobacco cessation efforts and promote healthier societies.
